# Nonlinear relationship between serum LH levels on hCG trigger day and live birth rates in patients with fresh embryo transfer: a retrospective cohort study

**DOI:** 10.3389/fendo.2026.1957191

**Published:** 2026-10-12

**Authors:** Yue Zhang, Yuxuan Xiao, Aimin Yang, Yang Yang, Lei Jiang, Jiahua Zheng, Kaixuan Guo, Wei Wang

**Affiliations:** 1 Hebei Key Laboratory of Infertility and Genetics, Department of Reproductive Medicine, Hebei Clinical Research Center for Birth Defects, Second Hospital of Hebei Medical University, Shijiazhuang, China; 2 Department of Obstetrics and Gynecology, The Second Hospital of Hebei Medical University, Shijiazhuang, China

**Keywords:** assisted reproductive technology, fresh embryo transfer, hCG trigger day, live birth rates, luteinizing hormone

## Abstract

**Background:**

In assisted reproductive technology (ART), the assessment of sex hormones is indispensable. Maintaining an appropriate level of luteinizing hormone (LH) is crucial for ovarian and endometrial development. However, the relationship between LH concentrations on the hCG trigger day and pregnancy outcomes after fresh embryo transfer (ET) remains relatively unclear. The aim of our study was to explore the role of serum LH levels in live birth rates (LBRs) and its specific relationship.

**Methods:**

We conducted a retrospective analysis of 9,044 patients who underwent fresh embryo transfer from 2019 to 2024. A multivariate regression model was used to identify the relationship. Stratification analysis was performed to test for effect modification in subgroups. The generalized additive model (GAM) and two-piecewise model were employed to describe the nonlinear relationship.

**Results:**

We found a positive relationship between serum LH levels and LBRs when LH concentrations were lower than 4.31 mIU/ml (odds ratio [OR] = 1.06, 95% confidence interval [CI] = 1.00-1.12, P = 0.042). No significant relationship was observed when LH concentrations were higher than 4.31 mIU/ml. In the exploratory subgroup analyses, the similar association was identified in GnRH agonist cycles, with an inflection point at 4.09 mIU/mL. The significant interaction was also identified between infertility type and serum LH levels in relation to LBRs.

**Conclusions:**

Our research found the nonlinear relationship between serum levels on the hCG trigger day and LBRs. We tentatively identified an inflection point at 4.31 mIU/ml, and further studies are warranted to validate its potential clinical utility.

## 1 Introduction

Assisted reproductive technology (ART) has become integral to infertility treatment, and controlled ovarian hyperstimulation (COH) markedly increases the number of maturing and ovulated follicles (1). During COH, endogenous luteinizing hormone (LH) secretion can be inhibited, which can prevent the premature LH surge. It is well known that LH exerts its effects by binding to LH/choriogonadotropin receptor (LHCGR) on ovary and glandular epithelium of endometria (2, 3). LH is essential for follicle development, ovulation and steroidogenesis, while also exerting regulatory effects on luteal function and endometrial growth (4–7). Therefore, precisely evaluating an individual’s hormone levels is a key prerequisite for achieving better outcomes (8).

The “luteinizing hormone clinical treatment window” theory has been introduced in some research (9). Maintaining an appropriate level of LH during COH is indispensable, as abnormal LH levels may result in inadequate endometrial hyperplasia, thereby affect pregnancy outcomes (10, 11). LH could be evaluated during the early, mid, and late follicular phases, as well as on the trigger day. In conjunction with basal LH levels, LH levels on hCG trigger day could also be beneficial in predicting the embryo development and pregnancy outcomes in women with polycystic ovary syndrome (PCOS) (12). Previous studies revealed that LH levels on hCG trigger day were positively associated with live birth rates (LBRs) in patients receiving the GnRH agonist and antagonist protocol (13, 14). While high levels of LH may indicate poor pregnancy outcomes in diminished ovarian reserve patients (15). The role of LH levels on hCG trigger day plays in predicting pregnancy outcomes remains relatively unclear and controversial.

Given the conflicting evidence, there is a growing interest in the impact of LH levels on the day of hCG trigger on live birth rates. This study aims to investigate whether the LH level on the day of hCG trigger is an independent factor affecting live birth rates in fresh cycles, and to illustrate the specific relationship.

## 2 Materials and methods

### 2.1 Study design and participants

The population of this retrospective cohort study comprised all autologous *in vitro* fertilization (IVF) and intracytoplasmic sperm injection (ICSI) cycles performed from January 1, 2019 to December 31, 2024. We restricted our analysis to fresh embryo transfer cycles to assess the direct association between LH levels on hCG trigger day and live birth rate. Cycles without fresh embryo transfer were excluded for the following reasons: 1) freeze-all strategy due to high OHSS risk; 2) absence of transferable embryos after fertilization and embryo culture; 3) suboptimal endometrial status or elevated progesterone on the hCG trigger day; 4) patient preference for deferred frozen embryo transfer. A detailed flow chart of the study population is presented in **Figure 1**. This study was approved by the ethics committee of the Second Hospital of Hebei Medical University, which granted a waiver of informed consent.

**Figure 1:**
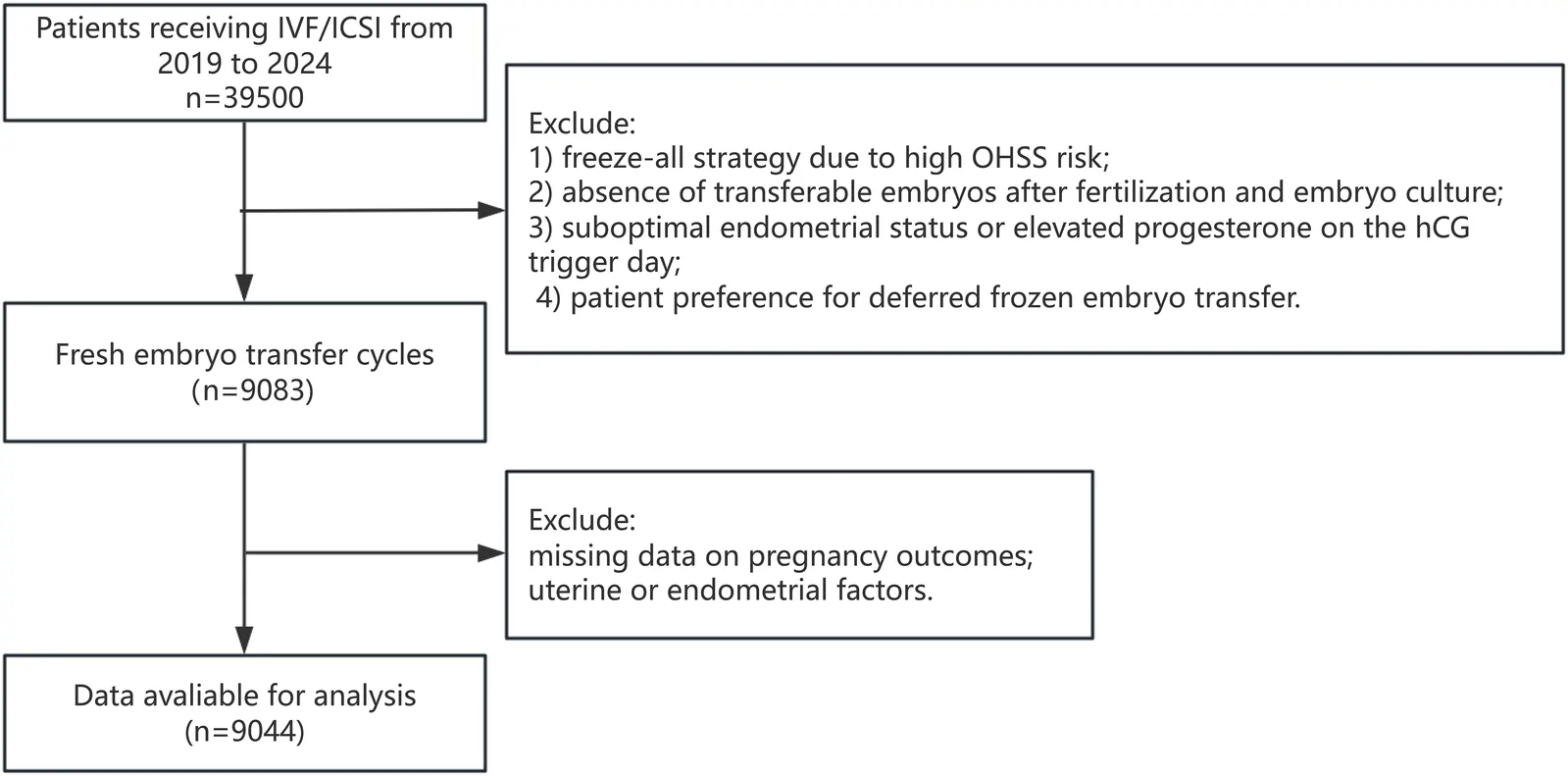
Detailed flow chart of the study population.

### 2.2 Ovarian stimulation

The ovarian stimulation in fresh cycles was achieved with combination of gonadotropin releasing hormone antagonist (GnRH-ant), agonist (GnRH-a) and recombinant follicle stimulating hormone (FSH). The doses were adjusted according to ovarian response assessed by serial ultrasound and measurement of serum hormone levels.

The GnRH antagonist protocol began on the day 2 or 3 of menstruation. Recombinant FSH at a dose of 100 to 300 IU were administered daily until the hCG trigger day. Patients undergoing the GnRH agonist protocol received 0.05 or 0.1 mg/day of Decapeptyl (Ferring AG, Dübendorf, Switzerland) starting in the mid-luteal phase. After approximately 14 days, once the down-regulation criteria were met, gonadotropin administration began and continued until the hCG trigger day. Under the prolonged GnRH agonist protocol, patients received 3.75 mg of triptorelin (Ipsen Pharma Biotech, Signes, France) on the second day of the menstrual cycle, followed by gonadotropin administration 28–31 days after achieving pituitary-ovarian suppression.

Oocyte maturation was induced with either 250 µg recombinant choriogonadotropin alfa (MerckSerono S.p.A., Geneva, Switzerland) or 8000–10,000 IU hCG (Lishenbao, Livzon Pharmaceutical Co., Ltd.) when at least three follicles ≥17 mm or at least one follicle ≥18 mm in diameter were observed in both ovaries. Serum LH levels were measured on the morning of hCG trigger day, prior to trigger medication administration. Venous blood samples were centrifuged immediately and assayed on the same day using an electrochemiluminescence immunoassay on a single analytical platform. The same instrument, reagent kit and assay protocol were used consistently throughout the study period. The intra-assay and inter-assay coefficients of variation for LH were <5% and <8%, respectively.

Oocyte retrieval was then performed under transvaginal ultrasound guidance 36–37 hours later. If the endometrium was in good condition (with a thickness of at least 8 mm and acceptable morphology) and there were no contraindications for transfer, a fresh embryo transfer might be performed on the 3rd to 5th day after retrieval. According to the consensus, 8% progesterone sustained-release vaginal gel (Xenoto, Merck Serono, Germany) and oral contraceptive tablets (10 mg twice daily) were used for luteal phase support, starting from the day of oocyte retrieval until 14 days after embryo transfer (ET).

### 2.3 Outcome

Biochemical pregnancy diagnosis was defined as a β-hCG ≥5 IU/L on day 14 after embryo transfer. Clinical pregnancy was identified as the presence of a gestational sac detected by ultrasound examination performed 30 days after embryo transfer. The delivery of at least one living infant was defined as live birth.

### 2.4 Statistical analysis

When analyzing the baseline characteristics among three groups, the method of mean ± SD, analysis of ANOVA (normally distributed variables) or Kruskal–Wallis tests (non-normally distributed variables) were applied to the continuous data. When comparing differences between two groups, we utilized t-test (normally distributed continuous variables) or Mann-Whitney (non-normally distributed continuous variables). While the number of cases (N), occurrence percentage (%) and Chi-square tests were used for categorical variables.

To address the non-independence of multiple cycles from the same patient, the generalized estimating equation (GEE) model was used to construct univariable and multivariable regression models for the prediction of live birth. Covariates were predefined based on clinical relevance and screened using the 10% change-in-estimate criterion. Collinearity was subsequently assessed using the variance inflation factor (VIF). Three models were established: unadjusted crude regression estimation; model I, estimates adjusted for female age, anti-Müllerian hormone (AMH) levels and antral follicle count (AFC); model II, estimates adjusted for all covariates (female age, body mass index (BMI), AMH levels, basal FSH level, basal LH level, stimulation protocol, the hCG level used on the trigger day, E2 level on hCG trigger day, P level on hCG trigger day, endometrium thickness on hCG trigger day). To describe the nonlinear relationship between the LH levels and live birth rates, the generalized additive model (GAM) and smooth plot were employed to characterize the curvilinear relationship. To quantify associations, we calculated crude odds ratios, adjusted OR and their corresponding 95% confidence intervals (CI).

To fit each interval in the nonlinear relationship, a two-piecewise linear model was adopted. The inflection point, where the derivative of a smooth curve approaches zero, was calculated using numerical differentiation. Bootstrap internal validation was used to evaluate the robustness of the inflection point. Stratification analysis was conducted based on female age, infertility type, BMI, AFC, AMH, stimulation protocol and method of fertilization.

## 3 Results

### 3.1 Demographic characteristics of patients

A total of 9044 fresh embryo transfer cycles were included in our study. **Table 1** presents the baseline characteristics of the patients enrolled. We analyzed ovarian stimulation parameters, embryo parameters, and baseline clinical characteristics. Patients with higher LH levels on the trigger day exhibited several notable characteristics: they were older, had lower AMH levels and AFCs. In terms of ovarian stimulation protocols, most participants with low LH levels used the GnRH antagonist protocol. In contrast, most patients with high LH levels used the GnRH agonist protocol, accounting for 74.1% of this cohort. The mean number of oocytes retrieved was 7.4 ± 4.4 in the high LH levels group, which was significantly lower compared with the low and middle LH levels groups. The pregnancy outcomes are showed in **Table 2**. The biochemical pregnancy rate (p = 0.0016), clinical pregnancy rate (p = 0.008) and live birth rate (p < 0.001) were all significantly lower in patients with elevated LH levels on the hCG trigger day. **Table 3** demonstrates the difference of parameters between cycles with live birth or not. The differences on age, infertility type, basal LH levels, AMH levels, AFC, stimulation protocol, sex hormone levels and endometrial thickness on the hCG trigger day, number of oocytes retrieved and embryo stage were statistically significant.

**Table 1:** Demographic characteristics of the participants.

Demographic characteristics	Serum LH Levels on hCG Trigger Day			P value
	Tertile1:<1.56	Tertile2:1.56-2.88	Tertile3:>2.88	
N	3023	3012	3009	
Female age(years)	31.4 ± 4.0	31.9 ± 4.1	33.0 ± 4.5	<0.001
Male age(years)	31.9 ± 4.1	32.4 ± 4.3	33.4 ± 4.6	<0.001
Infertility type				<0.001
Primary infertility	1511 (50%)	1447 (48%)	1346 (44.7%)
Secondary infertility	1512 (50%)	1565 (52%)	1663 (55.3%)
Infertility duration(years)	4.1 ± 2.6	4.0 ± 2.7	4.1 ± 2.7	0.736
BMI (kg.m.2)	23.8 ± 3.4	23.4 ± 3.2	23.8 ± 3.3	<0.001
Basal FSH (mIU/ml)	7.2 ± 1.9	7.5 ± 2.1	8.0 ± 2.4	<0.001
Basal E2 (pg/ml)	41.5 ± 23.3	42.6 ± 23.3	43.4 ± 23.8	0.008
Basal P(pg/ml)	0.7 ± 0.4	0.7 ± 0.4	0.7 ± 0.4	0.917
Basal PRL	13.6 ± 5.6	13.6 ± 5.7	13.4 ± 5.5	0.375
Basal LH (mIU/ml)	4.6 ± 2.5	4.6 ± 2.3	4.9 ± 2.4	<0.001
AMH	3.0 ± 1.7	2.6 ± 1.6	2.2 ± 1.7	<0.001
AFC	12.6 ± 5.6	11.3 ± 5.3	10.4 ± 5.8	<0.001
Stimulation protocol				<0.001
GnRH-agonist protocol	779 (25.8%)	1147 (38.1%)	2230 (74.1%)
GnRH-antagonist protocol	2241 (74.1%)	1833 (60.9%)	747 (24.8%)
Other protocols	3 (0.1%)	32 (1.1%)	32 (1.1%)
Gonadotropin dose (IU)	2698.3 ± 771.8	2525.8 ± 742.4	2478.8 ± 735.9	<0.001
The hCG level used on the trigger day (IU)	1846.9 ± 3396.2	2243.4 ± 3598.9	2305.7 ± 3610.5	<0.001
E2 level on hCG trigger day (pg/ml)	2712.4 ± 1449.6	2650.2 ± 1378.5	2164.3 ± 1425.5	<0.001
LH level on hCG trigger day (mIU/ml)	1.1 ± 0.3	2.1 ± 0.3	4.8 ± 1.6	<0.001
P level on hCG trigger day (pg/ml)	1.1 ± 0.5	1.0 ± 0.4	1.0 ± 0.4	<0.001
Endometrium thickness on hCG trigger day (mm)	11.0 ± 1.8	11.0 ± 1.8	10.7 ± 1.7	<0.001
Endometrial pattern on hCG trigger day				0.968
A	2057 (68%)	2054 (68.2%)	2059 (68.4%)
B	946 (31.3%)	940 (31.2%)	928 (30.8%)
C	20 (0.7%)	18 (0.6%)	22 (0.7%)
Number of oocytes retrieved	10.7 ± 4.6	9.5 ± 4.5	7.4 ± 4.4	<0.001
Number of embryos transferred	1.8 ± 0.4	1.8 ± 0.4	1.8 ± 0.4	<0.001
Embryo stage				0.091
Cleavage stage	2876 (95.1%)	2884 (95.8%)	2897 (96.3%)
Blastocyst stage	147 (4.9%)	128 (4.2%)	112 (3.7%)
Method of fertilization				0.002
IVF	2097 (69.4%)	2074 (68.9%)	2134 (70.9%)
ICSI	826 (27.3%)	876 (29.1%)	814 (27.1%)
IVF&ICSI	100 (3.3%)	62 (2.1%)	61 (2%)

The method of mean ± SD, analysis of ANOVA (normally distributed variables) or Kruskal–Wallis tests (non-normally distributed variables) were applied to the continuous data. While the number of cases (N), occurrence percentage (%) and Chi-square tests were used for categorical variables. BMI, body mass index; FSH, follicle stimulating hormone; E2, estradiol; P, progesterone; PRL, prolactin; LH, luteinizing hormone; AMH, anti-Müllerian hormone; AFC, antral follicle count; IVF, in vitro fertilization; ICSI, intracytoplasmic sperm injection.

**Table 2:** Pregnancy outcome of participants.

Pregnancy outcome	Serum Luteinizing hormone Levels on hCG Trigger Day			P value
	Tertile1:<1.60	Tertile2:1.60-2.79	Tertile3:>2.79	
Biochemical pregnancy				
N (%)	1529 (50.6%)	1545 (51.3%)	1438 (47.8%)	0.016
OR (95%CI)	1	1.03(0.93,1.14)	0.94 (0.81,0.99)	
Clinical pregnancy				0.008
N (%)	1424 (47.1%)	1453 (48.2%)	1335 (44.4%)	
OR (95%CI)	1	1.05 (0.95,1.16)	0.90 (0.81,0.99)	
Live birth				<0.001
N (%)	1081 (35.8%)	1082 (35.9%)	929 (30.9%)	
OR (95%CI)	1	1.01 (0.91,1.12)	0.80 (0.72,0.89)	

**Table 3:** Demographic characteristics of the participants stratified by outcome.

Demographic characteristics	Live birth	No live birth	P value
Female age(years)	31.2 ± 3.8	32.6 ± 4.4	<.001
Male age(years)	31.7 ± 4.0	33.0 ± 4.6	<.001
Infertility type			<.001
Primary infertility	1596 (51.6%)	2708 (45.5%)	
Secondary infertility	1496 (48.4%)	3244 (54.5%)	
Infertility duration(years)	4.0 ± 2.6	4.1 ± 2.7	0.016
BMI (kg.m.2)	23.7 ± 3.3	23.7 ± 3.3	0.583
Basal FSH (mIU/ml)	7.6 ± 2.1	7.6 ± 2.2	0.970
Basal E2 (pg/ml)	42.0 ± 22.9	42.7 ± 23.7	0.199
Basal P(pg/ml)	0.7 ± 0.4	0.7 ± 0.4	0.166
Basal PRL	13.5 ± 5.6	13.5 ± 5.6	0.764
Basal LH (mIU/ml)	4.8 ± 2.4	4.6 ± 2.3	<.001
AMH	2.9 ± 1.7	2.4 ± 1.6	<.001
AFC	12.2 ± 5.7	11.1 ± 5.6	<.001
Stimulation protocol			<.001
GnRH-agonist protocol	1201 (38.8%)	2955 (49.6%)
GnRH-antagonist protocol	1885 (61%)	2936 (49.3%)
Other protocols	6 (0.2%)	61 (1%)
Gonadotropin dose (IU)	2528.8 ± 742.0	2588.1 ± 762.5	<.001
The hCG level used on the trigger day (IU)	1972.8 ± 3422.7	2214.1 ± 3599.7	0.002
E2 level on hCG trigger day (pg/ml)	2736.4 ± 1420.5	2391.3 ± 1434.6	<.001
LH level on hCG trigger day (mIU/ml)	2.5 ± 1.7	2.7 ± 1.9	<.001
P level on hCG trigger day (pg/ml)	1.1 ± 0.4	1.0 ± 0.4	<.001
Endometrium thickness on hCG trigger day (mm)	11.1 ± 1.7	10.8 ± 1.8	<.001
Endometrial pattern on hCG trigger day			<.001
A	2225 (72%)	3945 (66.3%)
B	853 (27.6%)	1961 (32.9%)
C	14 (0.5%)	46 (0.8%)
Number of oocytes retrieved	10.0 ± 4.6	8.8 ± 4.7	<.001
Number of embryos transferred	1.9 ± 0.3	1.8 ± 0.4	<.001
Embryo stage			0.005
Cleavage stage	2986 (96.6%)	5671 (95.3%)
Blastocyst stage	106 (3.4%)	281 (4.7%)
Method of fertilization			0.330
IVF	2170 (70.2%)	4135 (69.5%)
ICSI	838 (27.1%)	1678 (28.2%)
IVF&ICSI	84 (2.7%)	139 (2.3%)

T-test (normally distributed continuous variables) or Mann-Whitney (non-normally distributed continuous variables) were used to compare between two groups. While the number of cases (N), occurrence percentage (%) and Chi-square tests were used for categorical variables.

### 3.2 Multivariate regression and stratification analysis

We performed the multivariate regression analysis to explore the relationship between LH levels on trigger day and LBRs. The results are presented in **Table 4**. In the unadjusted (crude) model, we observed a negative relationship between serum LH levels and LBRs (OR 0.93, 95% CI 0.91–0.96, P < 0.001). After adjusting for female age, AMH levels and AFC, the OR was 0.97 (95% CI 0.95–1.00, P = 0.02), indicating a persistently negative relationship. However, after adjusting for all corresponding covariates, the relationship was no longer statistically significant. Further adjustment for post-exposure embryonic variables in the sensitivity analysis did not materially alter the estimate (OR = 1.01, 95% CI: 0.98–1.04, P = 0.394) (**Supplementary Table S1**).

**Table 4:** Multivariable regression analysis models examining the association between LH level on hCG trigger day and LBRs.

LBR	Crude Model OR (95%CI)	P value	Model I OR (95%CI)	P value	Model II OR (95%CI)	P value
Serum LH Levels on hCG Trigger Day	0.93 (0.91,0.96)	<0.001	0.97 (0.95,1.00)	0.02	1.01 (0.98,1.04)	0.618
Patients were equally divided into three groups according to LH levels						
Low	1		1		1	
Intermediate	1.01 (0.91,1.12)	0.89	1.09 (0.98,1.21)	0.14	1.12 (1.00,1.25)	0.041
High	0.80 (0.72,0.89)	<0.001	0.95 (0.85,1.07)	0.41	1.10 (0.98,1.25)	0.117

Model I: adjusted for female age, AMH levels and AFC. The generalized estimating equation model was applied as the adjusted Model I.

Model II: following factors were adjusted: female age, BMI, AMH levels, basal FSH level, basal LH level, stimulation protocol, the hCG level used on the trigger day, E2 level on hCG trigger day, P level on hCG trigger day, endometrium thickness on hCG trigger day. The generalized estimating equation model was applied as the adjusted Model II.

As shown in **Table 5**, the stratification analysis was conducted in an exploratory manner. The results showed that there were interaction effects between infertility type and serum LH levels (P = 0.005). Although the association point estimates were in opposite directions in primary and secondary infertility subgroups, neither subgroup demonstrated a statistically significant association individually (primary infertility: OR = 1.04, P = 0.07; secondary infertility: OR = 0.98, P = 0.27). Compared with patients under 35 years, those over 35 years exhibited a different effect of LH levels on LBRs, although the interaction was not statistically significant (OR 0.96 vs 1.02, P = 0.051). Other factors failed to influence the relationship between LH levels and LBRs.

**Table 5:** Stratification analysis in different subgroups.

Stratification characteristics	N	LBR		P for interaction
		Adjusted OR (95%Cl)	P value	
Female age				0.051
≤35	6977	1.02 (0.99,1.06)	0.187	
>35	2067	0.96 (0.90,1.02)	0.212	
Infertility type				0.005
Primary infertility	4304	1.04 (1.00,1.08)	0.07	
Secondary infertility	4740	0.98 (0.94,1.02)	0.27	
BMI (kg/m2)				0.383
≤24	5298	1.02 (0.98,1.06)	0.363	
>24	3746	0.99 (0.95,1.04)	0.765	
AMH				0.595
≤1.2ng/ml	1938	0.98 (0.93,1.04)	0.468	
>1.2ng/ml	7106	1.02 (0.98,1.05)	0.334	
Method of fertilization				0.976
IVF	6305	1,00 (0,96,1.03)	0.872	
ICSI	2516	1,02 (0.97,1.08)	0.416	

The following factors, except the stratification factor itself, were adjusted in the multivariable analysis.

### 3.3 Nonlinear relationship between serum LH levels on hCG trigger day and LBRs

The smooth curve revealed an obvious nonlinear relationship between serum LH levels and LBRs after adjusting for all covariates (**Figure 2**). To describe this specific relationship, we utilized a two-piecewise linear model (**Table 6**). The inflection point was determined to be 4.31 mIU/ml LH using numerical differentiation. When the concentration of LH was lower than 4.31 mIU/ml, LBRs increased with rising LH levels (OR 1.06, 95% CI 1.00-1.12, P = 0.042). Conversely, when the concentration of LH was higher than 4.31 mIU/ml, serum LH levels on the hCG trigger day showed no statistically significant association with LBRs (OR 0.99, 95% CI 0.90-1.10, P = 0.903). In the bootstrap validation, the median inflection point from bootstrap samples was 4.17 mIU/mL (95% percentile CI: 2.67–5.58 mIU/mL), which was close to the original inflection point. The effect estimate was robust with minimal optimism bias (original OR 0.95, bootstrap OR 0.94), and most resampled datasets retained the same direction of association (**Supplementary Table S2**). We performed an exploratory analysis to examine whether stimulation protocols modify the association between LH levels on hCG trigger day and live birth rate (**Table 7**). The overall interaction was not statistically significant (P = 0.255). We further investigated this relationship separately within GnRH agonist and GnRH antagonist cycles. The nonlinear association was identified in GnRH agonist cycles, where live birth rate was positively correlated with LH levels below 4.09 mIU/mL (OR 1.09, 95% CI 1.00-1.20, P = 0.048) (**Figure 3**). In contrast, no statistically significant association was observed in GnRH antagonist cycles (**Figure 4**).

**Figure 2:**
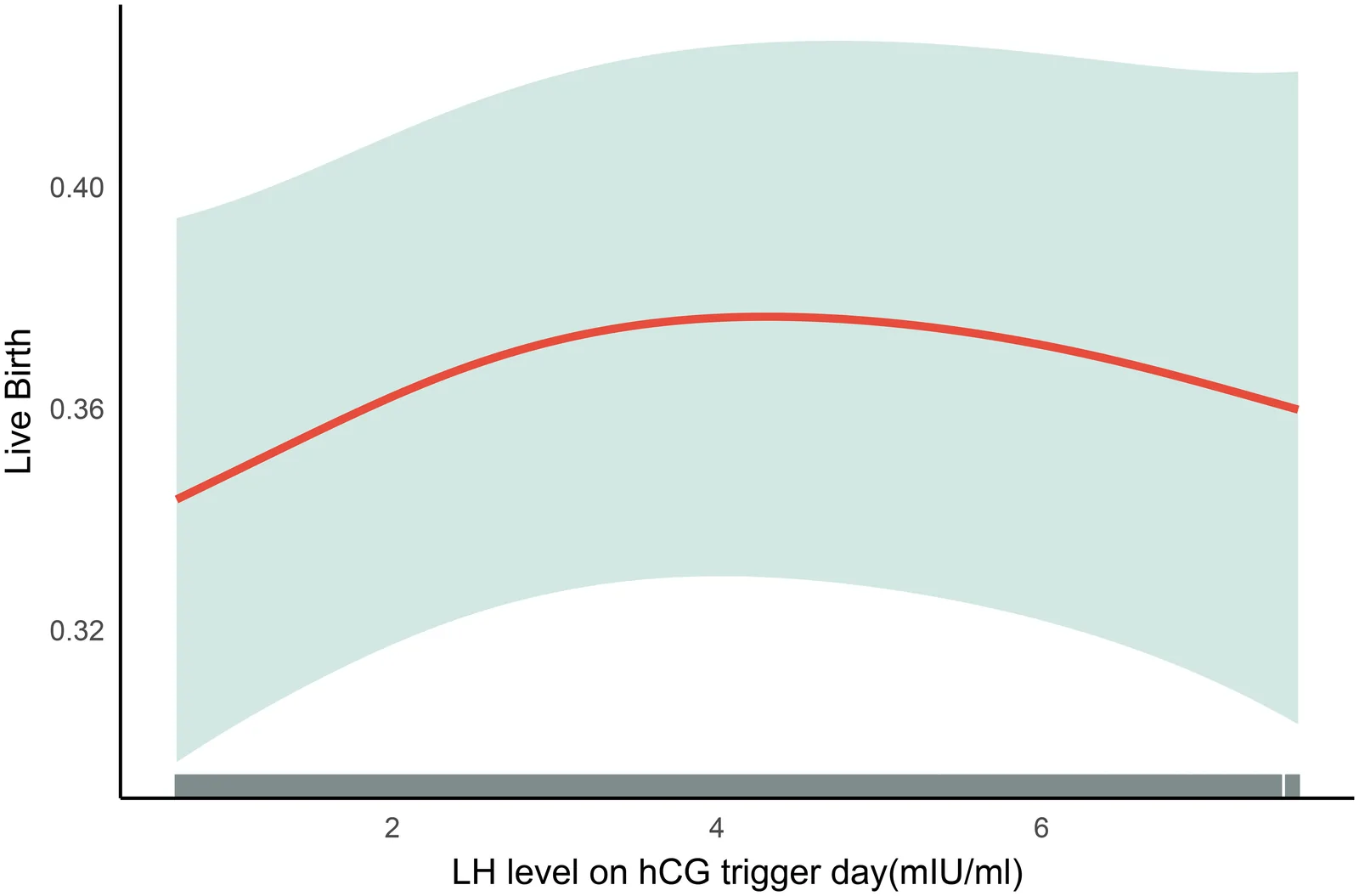
Association between serum LH level on the hCG trigger day and live birth rates. A nonlinear association between live birth and LH level was found in a generalized additive model (GAM). Solid red line represents the smooth curve fit between these two variables. Green lines represent the 95% of confidence interval from the fit.

**Table 6:** The result of two-piecewise linear regression model.

Models	Adjusted OR (95%CI)	P value
Model I		
One line slope	1.01 (0.98,1.04)	0.618
Model II		
Inflection point	4.31	
<4.31	1.06 (1.00,1.12)	0.042
>4.31	0.99 (0.90,1.10)	0.903

Adjustment variables: female age, BMI, AMH levels, basal FSH level, basal LH level, stimulation protocol, the hCG level used on the trigger day, E2 level on hCG trigger day, P level on hCG trigger day, endometrium thickness on hCG trigger day.

**Table 7:** Regression models stratified by GnRH agonist and antagonist protocols.

	Adjusted OR (95%CI)	P value
LH levels*Stimulation protocol	1.04(0.97,1.11)	0.255
GnRH agonist protocol		
Model I		
One line slope	1.00(0.97,1.03)	0.934
Model II		
<4.09	1.09(1.00,1.20)	0.048
>4.09	1.03(0.93,1.14)	0.577
GnRH antagonist protocol		
Model I		
One line slope	1.04(0.98,1.10)	0.2
Model II		
<4.49	1.06(0.99,1.14)	0.096
>4.49	0.74(0.51,1.06)	0.101

Adjustment variables: female age, BMI, AMH levels, basal FSH level, basal LH level, stimulation protocol, the hCG level used on the trigger day, E2 level on hCG trigger day, P level on hCG trigger day, endometrium thickness on hCG trigger day.

**Figure 3:**
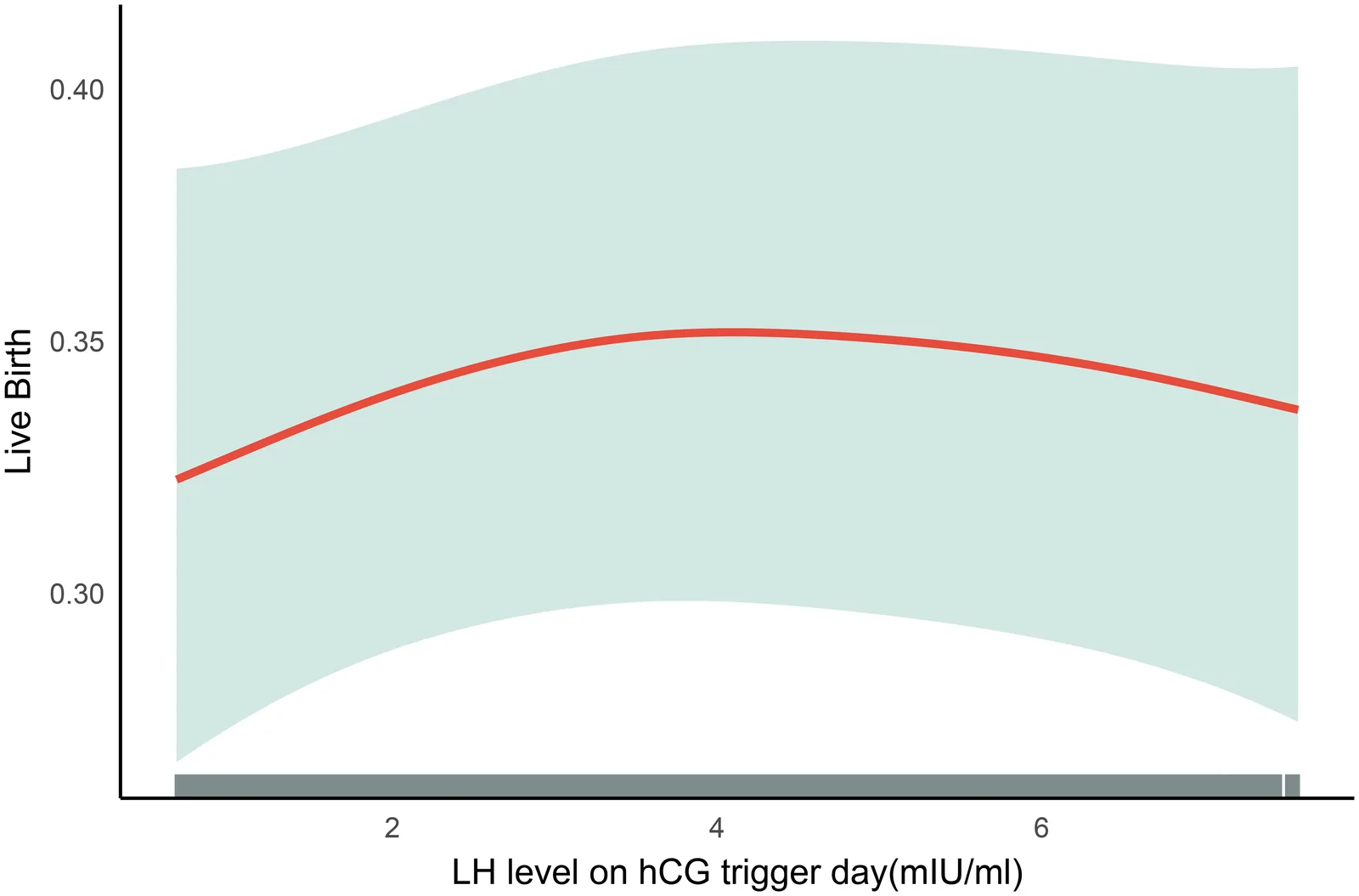
Association between serum LH level on the hCG trigger day and live birth rates in GnRH agonist cycles. A nonlinear association between live birth and LH level was found in a generalized additive model (GAM) in GnRH agonist cycles. Solid red line represents the smooth curve fit between these two variables. Green lines represent the 95% of confidence interval from the fit.

**Figure 4:**
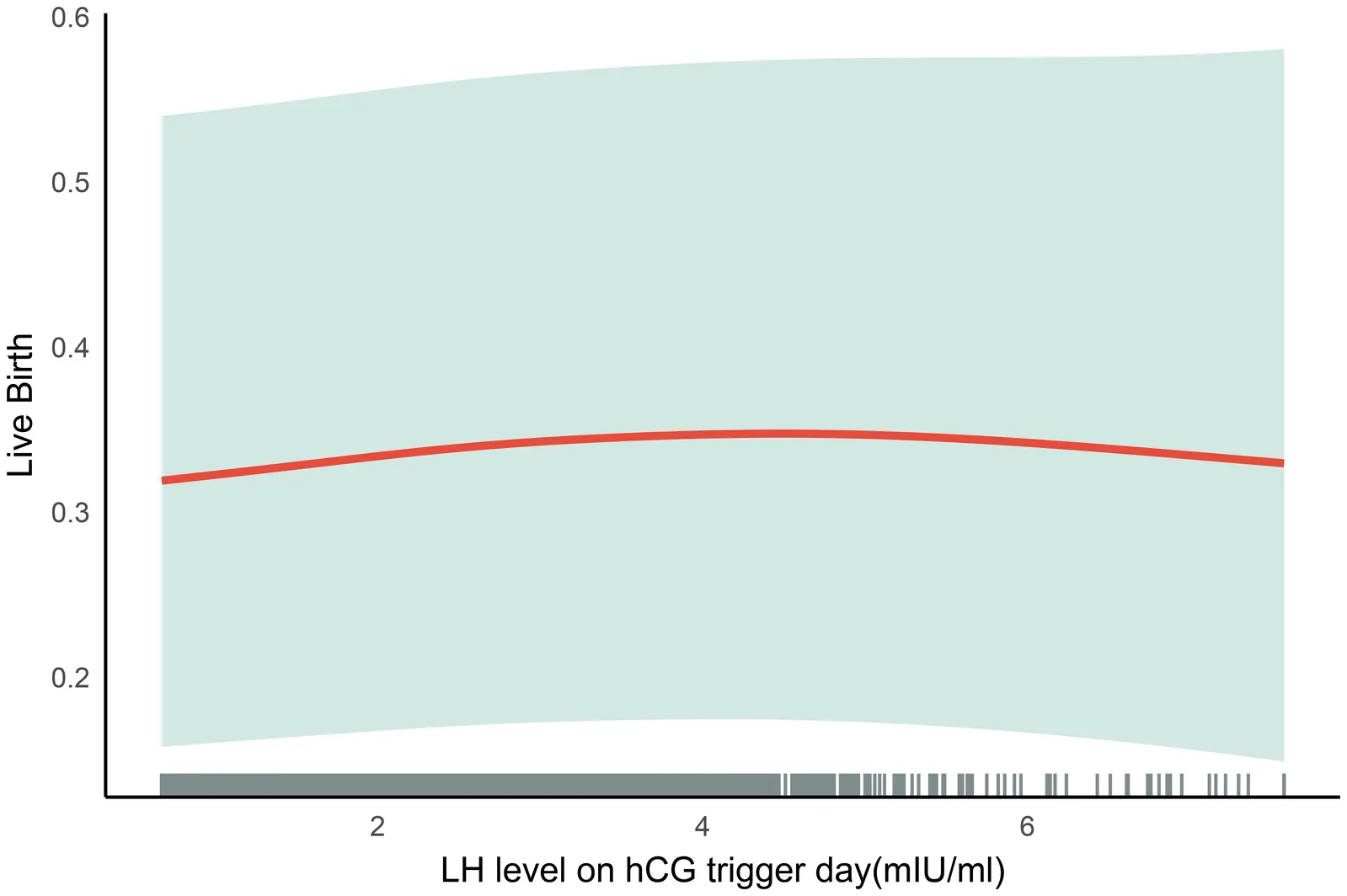
Association between serum LH level on the hCG trigger day and live birth rates in GnRH antagonist cycles. Solid red line represents the smooth curve fit between these two variables. Green lines represent the 95% of confidence interval from the fit.

## 4 Discussion

Our research found the nonlinear relationship between serum LH levels on the hCG trigger day and LBRs. The two-piecewise model revealed a positive relationship between LH concentrations and LBRs when LH concentrations were below 4.31 mIU/ml. In contrast, no significant relationship was observed when LH concentrations exceeded 4.31 mIU/ml.

In the generalized additive model and smooth plot, we preliminarily identified an inflection point. We found the LBRs increased by about 6% with every 1mIU/ml increase in LH when LH concentrations were lower than 4.31 mIU/ml. In the exploratory subgroup analyses, we found the nonlinear relationship was only identified in GnRH agonist cycles. Some previous studies were consistent with our study. In a study conducted in 2021,LH was found to be the protective factors for live births in GnRH agonist regimen of norm gonadotrophic women, with OR of 1.696 (95% CI: 1.345-2.139) (16). Another study observed that in patients undergoing fresh embryo transfer, those with insufficient LH levels on the trigger day exhibited reduced pregnancy and live birth rates, along with increased early miscarriage rates (13). Zhou et al. analyzed GnRH antagonist cycles among patients with normal ovarian reserve and PCOS, and reported that elevated serum LH levels were associated with higher LBRs following fresh embryo transfer (14). In contrast to their findings, we observed no significant association in antagonist cycles. Further investigations are warranted to reconcile these discrepant results.

Successful implantation is contingent upon the precise synchronization between embryo development and endometrial receptivity. In ovary, LH receptors are primarily expressed in the zona pellucida of oocytes, granulosa cells, and luteal cells (17). LH can directly affect oocyte quality, embryo growth and implantation, as well as luteal function (18, 19). Earlier studies have indicated that low LH concentrations on hCG trigger day correlate with diminished mature oocyte yield and impaired live birth rates (13, 20, 21). Additionally, functional LH receptor on the endometrium has been found in human studies (22, 23), which is regulated by the menstrual cycle and presents periodic changes. Its role is mainly to stimulate uterine growth, proliferation, and diastolic uterine movement (24). Non-physiological hormone levels during COH disrupted embryo-endometrial synchrony, impairing the receptivity of the endometrium (25, 26). Notably, LH might play an important role in regulating the endometrium during the implantation window (27). Low endogenous LH levels were associated with low endometrial maturity in IVF cycles (28), but not the thickness of the endometrium (29). Additionally, a transcriptome analysis conducted by Shuo Han revealed that LH can modulate the expression of key gene and downstream signaling pathways in endometrium (30). LH also participates in immune regulation in the endometrium via regulating the function of B and T cells (31). Low LH levels have a more pronounced impact on endometrial cell function compared to high LH levels.

LH levels should be maintained within an optimal range (9, 32). Our study revealed that once serum LH concentrations reached a specific threshold on the hCG trigger day, the positive correlation ceased to exist. Previous studies revealed that elevated LH levels may disturb the interaction between cumulus cells and granulosa cells, which in turn affects oocyte development (32). The embryo implantation rate and clinical pregnancy rate were reported significantly lower in patients with LH level on trigger day higher than 3.14 IU/L in POSEIDON group 4 (15). Another study demonstrated higher LH levels (≥ 32 IU/L) has been reported to lead to a decreased LBRs in frozen embryo transfer cycles when the P level is less than 1 ng/mL (33). Additionally, our stratified analysis revealed the interaction between infertility type and the association of serum LH levels with LBRs. Notably, this subgroup finding is exploratory in nature.

Our research has several limitations. First, our study may introduce selection bias, as patients who were unsuitable for fresh transfer were excluded. The findings cannot be generalized to all stimulated IVF/ICSI cycles. Second, the inflection point identified by the GAM model was data-derived from this cohort. Its clinical applicability requires further validation. Third, findings from protocol-specific and infertility-type subgroup analyses were explicitly exploratory and required replication in future studies.

## 5 Conclusions

Our study revealed the nonlinear relationship between serum LH levels on hCG trigger day and LBRs. A positive association was observed only when LH levels were lower than 4.31 mIU/ml. This finding underscores the clinical significance of monitoring serum LH levels before hCG administration. The observed inflection point of 4.31 mIU/mL is a hypothesis-generating finding. Further external validation and prospective studies with rigorous design are warranted to validate its value and explore its potential clinical application.

## Data Availability

The raw data supporting the conclusions of this article will be made available by the authors, without undue reservation.
