# Supplementary material for: Nonlinear relationship between serum LH levels on hCG trigger day and live birth rates in patients with fresh embryo transfer: a retrospective cohort study
Source: Front Endocrinol (Lausanne). 2026 Oct 12;17:1957191. doi: 10.3389/fendo.2026.1957191 (PMC13696257; doi:10.3389/fendo.2026.1957191)
Supplement: Supplementary file 1 [file Table1.docx]

| LBR | Adjusted OR (95%CI) | P value |
| --- | --- | --- |
| Model A | | |
| Serum LH Levels on hCG Trigger Day | 1.01 (0.98,1.04) | 0.618 |
| Model B | | |
| Serum LH Levels on hCG Trigger Day | 1.01 (0.98,1.04) | 0.394 |

Table S1. Sensitivity analysis of associations between LH level on hCG trigger day and LBR.

Model A: Adjusted for female age, BMI, AMH levels, basal FSH level, basal LH level, stimulation protocol, the hCG level used on the trigger day, E2 level on hCG trigger day, P level on hCG trigger day, endometrium thickness on hCG trigger day.

Model B: Adjusted for female age, BMI, AMH levels, basal FSH level, basal LH level, stimulation protocol, the hCG level used on the trigger day, E2 level on hCG trigger day, P level on hCG trigger day, endometrium thickness on hCG trigger day, number of oocytes retrieved, number of embryos transferred, method of fertilization and embryo stage.

| Parameter | Value |
| --- | --- |
| Bootstrap median inflection point | 4.17 |
| Bootstrap 95% percentile CI for inflection point | (2.67, 5.58) |
| Original regression coefficient (β) | −0.0566 |
| Original odds ratio (OR) | 0.95 |
| Median bootstrap β | −0.0626 |
| Median bootstrap OR | 0.94 |
| Bootstrap 95% CI for OR | (0.844, 1.041) |
| Valid convergent models | 1000 / 1000 |
| Proportion of resamples with consistent effect direction | 89.0% |

Table S2. The result of bootstrap internal validation results with the inflection point.
